# Prevention and treatment of pericardial tamponade in the electrophysiology laboratory: a European Heart Rhythm Association survey

**DOI:** 10.1093/europace/euad378

**Published:** 2024-01-02

**Authors:** Andreas Metzner, Arian Sultan, Piotr Futyma, Sergio Richter, Laura Perrotta, K R Julian Chun

**Affiliations:** Department of Cardiology, University Heart and Vascular Center Hamburg, Martinistr. 51, 20246 Hamburg, Germany; Department of Cardiology, University Heart Center Cologne, Cologne, Germany; St. Joseph’s Heart Rhythm Center Rzeszow and Medical College, University of Rzeszow, Rzeszow, Poland; Heart Center Dresden, University Hospital, Technical University Dresden, Dresden, Germany; Department of Cardiology, Careggi University Hospital, Florence, Italy; CCB, Agaplesion Markus Hospital, Frankfurt, Germany

**Keywords:** Electrophysiology, Pericardial tamponade, Complications, Treatment

## Abstract

**Aims:**

Pericardial tamponade (PT) is the most frequent severe complication during electrophysiology (EP) procedures and requires immediate, co-ordinated, and effective treatment. However, multiple aspects of PT treatment are either not standardized or are under ongoing debate.

**Methods and results:**

An online questionnaire consisting of 26 multiple-choice questions was sent out to the European Heart Rhythm (EHRA) Research Network and also distributed via social media outputs. The EHRA survey was conducted between May and June 2023. A total of 213 replies were received from European (87%) and non-European countries. Ninety per cent of all participants perform interventions in dedicated EP labs equipped with different ablation platforms. In case of PT, most participants use X-ray as the main imaging modality guiding pericardial puncture, predominantly aiming for an anterior puncture site. Sheaths of different sizes are introduced into the pericardial space (84.3%), followed by a pigtail catheter. Application of protamine is an established but variable step in the majority (84.6%). Novel oral anticoagulants (NOAC) antidotes are not used by 73.3% of participants, while 15.2% routinely apply them. Re-transfusion of aspirated blood is performed by 72.1% [before protamine administration (18.2%), after protamine administration (13.5%), if pericardial effusion cannot be controlled (40.4%)]. A total of 72.4% re-transfuse without blood filter systems. A decision for surgical intervention is mostly taken if bleeding continues despite all interventional measures.

**Conclusion:**

The current survey demonstrates that the management of PT is heterogeneous among centres. The findings of this survey may help to guide operators in their treatment and decisions in the setting of PT.

## Graphical Abstract

**Graphical Abstract:**
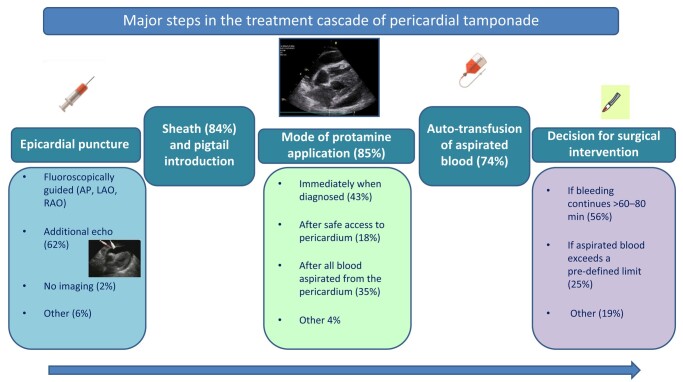

**What’s new?**

- Most centres have no restrictions regarding age and body mass index even for complex left atrial/ventricular procedures.
- Transseptal puncture is mostly performed fluoroscopically and frequently facilitated by diagnostic catheters and/or additional imaging modalities such as transoesophageal echocardiography or intracardiac echocardiography.
- In case of pericardial tamponade (PT), pericardial puncture is mainly guided by fluoroscopy and echocardiography and most responders aim for an anterior puncture site, followed by the introduction of a sheath and a pigtail catheter.
- Protamine is applied by a majority of participants immediately when PT is diagnosed or after complete drainage of pericardial effusion. Novel oral anticoagulants (NOAC) antidotes are administered only by a minority of respondents.
- A majority does directly auto-transfuse aspirated blood without a blood filter and with no maximal limit for blood re-transfusion.
- Surgical intervention is mainly considered if bleeding continues despite all interventional measures.

## Introduction

Catheter ablation is an established treatment option for various types of arrhythmias and, in general, has a high success rate and an excellent safety profile.^1^ However, despite increasing experience and improved ablation strategies and technologies, complications can occur and can be potentially life-threatening.^2–14^ Pericardial tamponade (PT) is the most frequent severe complication during electrophysiology (EP) procedures and requires immediate, co-ordinated, and effective treatment. However, the treatment of PT is not standardized and various aspects are still under debate.^15^ Therefore, we conducted a survey evaluating the infrastructure, safety precautions, and treatment strategies in the setting of PT in European and non-European EP centres.

## Methods

An online questionnaire consisting of 26 multiple-choice questions was sent to the European Heart Rhythm (EHRA) Research Network and was also distributed via social media platforms. The exact questionnaire is provided as Supplementary material online. The EHRA survey was conducted between May and June 2023.

## Results

### Baseline data

**Figure 1:**
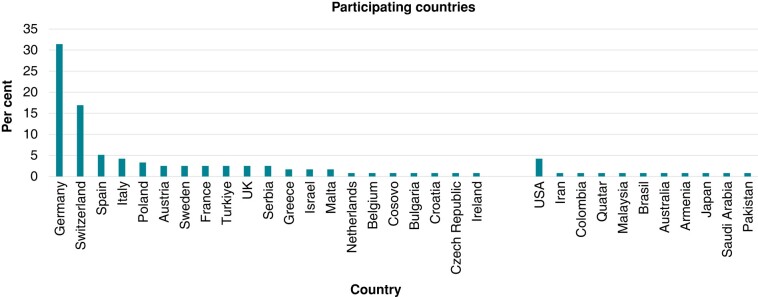
Participating countries.

**Figure 2:**
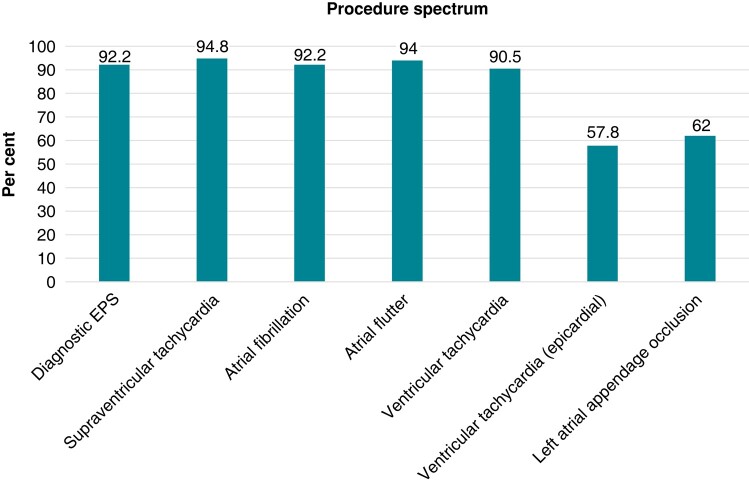
A spectrum of procedures.

We received a total of 213 replies. The majority of respondents were from European countries (87%) and 13% were from non-European countries (*Figure 1*). A total of 68% of all participants practice at academic institutions and 32% at non-academic hospitals. The number of ablation procedures per year varied from up to 500 EP procedures in 45% of participants, 501–1000 procedures in 27%, 1001–1500 procedures in 14%, and finally, more than 1500 procedures in the remaining 15%. While 92% of all participating EPs report to perform diagnostic EP-procedures, 95% perform ablations of supraventricular tachycardias, 92% of atrial fibrillation, 94% of atrial flutter, and 91% of ventricular tachycardia (VT). Of note, a total of 58% of responders perform epicardial VT ablation and 62% offer interventional occlusion of the left atrial appendage (*Figure 2*).

### Electrophysiology infrastructure and equipment

Reflecting the spectrum and load of EP procedures, 90.2% of all participants perform their interventions in dedicated EP labs. With 91%, three-dimensional (3D) mapping, in combination with irrigated contact force–enabled radiofrequency (RF) catheters, is the most frequently available ablation modality, followed by cryoballoon technology (79.5%). Conventional RF ablation is applied in (71.4%), and non-contact-force-guided RF ablation with 3D mapping in (44.6%). Of note, pulsed field ablation (PFA) is already widely spread and applied by 29.5% of all participants. Another 7.1% are also equipped with other ablation platforms.

About two-thirds (65.8%) of all responders have institutional cardiac surgery available on site. A total of 88.2% of the participants report to have echocardiography permanently available inside the EP lab. Moreover, 94.6% answered to have a pre-prepared epicardial puncture set inside the lab that contains all necessary items for emergency epicardial puncture and drainage.

### Pre-procedural considerations

While 72.7% of participants report to have no restrictions for the patient’s body mass index (BMI), 8.2% answered to have BMI limits for all procedures, and another 19.1% have BMI restrictions for left atrial and left ventricular ablation procedures only. Body mass index limits ranged between 35 and 55 kg/m^2^. With regard to age, 85.2% have no age limits, while 3.7% have age limits for all procedures, and 11.1% for left atrial and left ventricular interventions only (ranging from 75 to 85 years). International normalized ratio (INR) limits for patients on vitamin K-antagonists were reported by 32.7%, with INR limits ranging from 2 to 4. The remaining participants did not report INR restrictions for any EP procedures. A total of 20.9% of responders do not interrupt novel oral anticoagulants (NOAC) therapy, 15.5% stop it the day before, 18.2% the evening before, and 41.8% at the day of the procedure. A total of 3.6% report other strategies.

### Procedural aspects and safety considerations

A total of 1.8% of participants monitor blood pressure invasively during diagnostic EP procedures, 9% during ablation of atrial flutter or atrial tachycardia, 13.6% during ablation of atrial fibrillation, and 13.6% for interventional closure of the left atrial appendage. In VT ablation procedures, blood pressure is monitored invasively by 84.5%. Another 13.6% report to uniquely use non-invasive blood pressure monitoring for all procedures.

**Figure 3:**
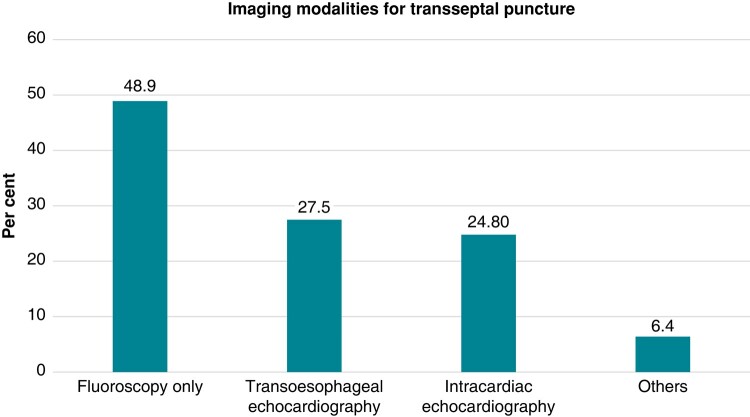
Imaging modalities for transseptal puncture.

Transseptal puncture is guided by fluoroscopy only in 48.9% of respondents. Transoesopageal echocardiography (TOE) as an additional imaging mode is used by 27.5% and intracardiac echocardiography (ICE) by 24.8% of the participants. A total of 48.9% purely rely on fluoroscopy and 6.4% report to use other guiding modalities for transseptal puncture (*Figure 3*). Diagnostic catheters are positioned within the coronary sinus by the majority of participants (86.1%). A total of 23.1% also place a diagnostic catheter at the His bundle region for transseptal puncture, and 8.3% use a pigtail catheter or wire inside the aorta. The remaining 9.3% report to use no diagnostic catheter for guidance of transseptal punctures.

Additional modalities used for transseptal puncture (TP) are pressure control (50.1%), contrast staining of the fossa ovalis before advancing the transseptal needle (29.4%), introduction of a guidewire into the left atrium or the left superior pulmonary vein once transseptal puncture is performed (55.9%), and/or contrast injection after transseptal access (49%), respectively (*Figure 3*).

### Treatment of pericardial tamponade

For pericardiocentesis of a PT, most participants report to use X-ray as the main imaging modality to guide pericardial puncture. The most frequently used views are anterior-posterior (58.7%) and left anterior oblique (45.2%). A total of 14.2% also perform the puncture in a right anterior oblique angulation (14.4%). In addition to X-ray, echocardiography is used by 61.5%. A minority of responders does not use any imaging modality and 5.8% report to use other modalities. Pericardial puncture can be targeted on the anterior and the posterior sites. An anterior access is preferred by most (67.3%). Once epicardial access is gained, most physicians (84.3%) introduce sheaths of different sizes into the pericardial space (5F 9.8%, 6F 35.3%, 7F 14.7%, 8F 21.6%, other sizes 2.9%), followed by a pigtail catheter (5F 23.5%, 6F 50%, 7F 19.6%, other sizes 6.7%).

**Figure 4:**
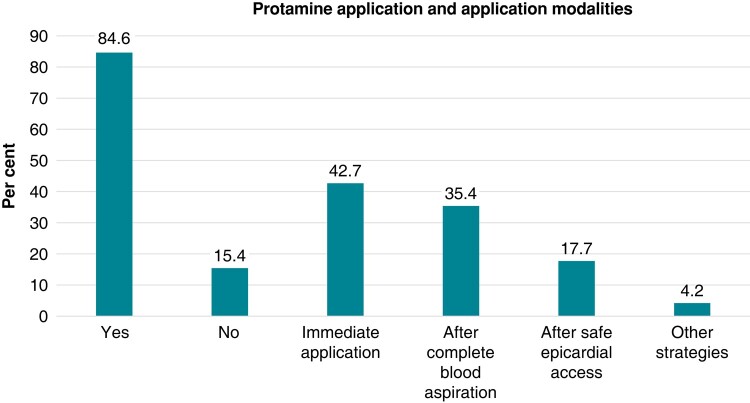
The application of protamine.

The majority of respondents (84.6%) applies protamine in case of a PT. The timing of protamine injection varies with injection immediately upon a diagnosis of PT in 42.7%, after complete drainage of PT in 35.4%, and after successful access to the pericardium in 17.7%. Some (4.2%) report other strategies regarding protamine application (*Figure 4*). The protamine dose is adjusted according to the last measured activated clotting time (ACT) level in 43.3%, and 37.1% apply protaminin at a ratio of 1:1 to previous heparin administration. Among the remaining participants, 3000 and 5000 I.E. are given as an institutional standard in 4.1 and 9.3%, respectively.

NOAC antidotes are routinely administered by 15.2% of respondents, while 73.3% of them never use antidotes. Another 11.4% apply NOAC antidotes only in certain situations such as unresponsiveness of bleeding to protamine administration. Additional application of clotting factors is not considered by 91.7% of centres. However, 8.3% would apply prothrombin complex, fresh frozen plasma, or tranexamic acid as necessary.

**Figure 5:**
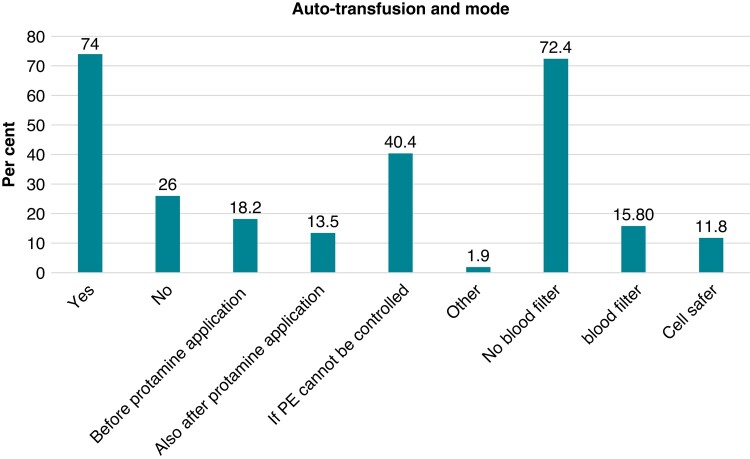
An auto-transfusion of aspirated blood and the mode of auto-transfusion.

Auto-transfusion of aspirated blood is reported to be done by 76% of all participants. Some start auto-transfusion before protamine administration (18.2%), others after protamine administration (13.5%), and others only if pericardial effusion cannot be controlled (40.4%). Another 1.9% report to have other strategies regarding re- or auto-transfusion. For auto-transfusion, 72.4% of the participants do not use a blood filter, 15.8% use a blood filter, and another 11.8% auto-transfuse via a cell safer only (*Figure 5*). While 90.4% do not have a maximal limit of re-transfused blood, 6.4% have defined limits (maximum of 1–2 L), and 3.2% have different strategies.

The decision for surgical intervention is mostly taken if bleeding continues despite all interventional measures. Accordingly, 55.7% of the participants answered to decide for surgical backup and intervention if the bleeding continues for more than 60–80 min. Another 25.4% consider surgical assistance if the amount of aspirated blood exceeds a predefined limit, ranging from 1000 to 3000 mL among centres. Another 18.9% consider other measures such as no reduction of aspirated blood per minute, haemodynamic instability, or a suspicion of a left atrial/ventricular defect.

### Post-interventional aspects

After successful epicardial puncture, drainage, and stabilization of the patient, most respondents (48.5%) keep the pigtail catheter until there is no evidence of further bleeding after re-initiation of an indicated anticoagulation. Another 7.9% remove the pigtail as soon as the bleeding stops, and 43.6% report other strategies such as keeping the pigtail for 2–72 h and including repeat echocardiography showing complete drainage of effusion without further aspiration.

Specific medications applied after PT drainage are non-steroidal anti-inflammatory drugs (NSAIDs) in 48.6% of participants for a mean of 10 days, colchicine in 47.2% for a mean of 19 days, cortisone in 8.3% as a single shot in the majority, and/or antibiotics in 31.9% for a mean of 2 days.

If indicated, anticoagulation therapy is re-initiated within 0–72 h after pigtail removal.

### Onsite cardiac surgery vs. no onsite cardiac surgery

While many aspects between centres with and without onsite cardiac surgery are comparable, there are also major differences. The mean total number of procedures in participant’s centres with onsite cardiac surgery is higher with 850 ± 595 vs. 634 ± 569. Operators at centres without cardiac surgery less frequently report to perform epicardia VT ablation (37 vs. 72%), and protamine is more often regularly applied in case of PT (81 vs. 70%).

## Discussion

Despite technological advancements and procedural expertise, PT remains a frequent and potentially life-threatening complication in the EP lab. However, in reputed centres and when managed by experienced operators, PT can be effectively treated. There are no general recommendations on how to prevent and how to treat PT. The current survey found the following:

1. Most centres have no restrictions regarding age and BMI even for complex left atrial/ventricular procedures.
2. Transseptal puncture is mostly performed fluoroscopically and is frequently facilitated by diagnostic catheters and/or additional imaging modalities such as TOE or ICE.
3. In case of PT, pericardial puncture is mainly guided by fluoroscopy and echocardiography, and most responders aim for an anterior puncture site, followed by the introduction of a sheath and a pigtail catheter.
4. Protamine is applied by a majority of participants immediately when PT is diagnosed or after complete drainage of pericardial effusion. NOAC antidotes are administered only by a minority of respondents.
5. A majority does directly auto-transfuse aspirated blood without a blood filter and with no maximal limit for blood re-transfusion.
6. Surgical intervention is mainly considered if bleeding continues despite all interventional measures.

### Electrophysiology-infrastructure and equipment

While two-thirds of all responders report to have institutional cardiac surgery, almost all state to have echocardiography permanently available inside the EP lab and prepared epicardial puncture sets. Although institutional cardiac surgery might extend the window for interventional measures to treat PT, echocardiography permanently on hand and prepared puncture sets allow for straightforward and time-efficient diagnosis and emergency treatment without unnecessary loss of time.

### Procedural aspects and safety considerations

Transseptal mispuncture is one of the main reasons for PT. Different techniques can be applied, and finally, the mode of transseptal puncture is influenced by individualized strategies and by personal experience. However, the ultimate demand is to perform transseptal punctures as controlled and as safe as possible. To facilitate transseptal puncture, many centres use catheters at different anatomical positions to improve understanding of the individual anatomy. A catheter inside the coronary sinus will provide a rough visualization of the mitral valve and left atrial dimensions. In addition, a synchronous movement of the transseptal sheath and transseptal needle assembly positioned at the fossa ovalis with the coronary sinus catheter indicates adequate septal contact and position. Some participants use an additional catheter at the His bundle region or a pigtail catheter or wire inside the aorta to mark the aortic root to prevent inadvertent aortic puncture.^16,17^ Mostly, transseptal puncture is guided by fluoroscopy only, but other imaging modalities might be added, such as transoesophageal or ICE. Both echo modalities will not only help to guide the transseptal sheath and needle to the fossa ovalis but will also facilitate to specifically target anterior or posterior puncture sites within the fossa ovalis depending on the ablation system used (e.g. cryoballoon ablation posterior and inferior, in PFA ablation rather midseptal and inferior) or the intended ablation strategy (e.g. pulmonary vein isolation or antegrade left ventricular access). Transseptal puncture with pressure control is applied by most operators. Verification of successful left atrial access before advancing the transseptal sheath by either introducing a guidewire into the left atrium or a pulmonary vein or injecting a contrast medium helps to avoid advancement of the sheath in case of inadvertent pericardial puncture.

### Treatment of pericardial tamponade

There are different ways to gain epicardial access, but two-thirds of all responders prefer an anterior epicardial puncture site that has been shown to be safer than a posterior one.^18^ In an analysis by Mathew *et al*.,^18^ a posterior epicardial access was strongly associated with a higher rate of severe puncture-related complications and a higher necessity for later surgical repair. Fluoroscopy in different views and additional echocardiography as answered by three out of four EPs are the leading imaging modalities to guide the puncture. Almost all participants introduce a sheath into the pericardial space as soon as access is established. A sheath has two major procedural advantages. First, it can be used for direct aspiration of blood, and the bigger the size, the more volume can be mobilized. Second, blood inside the pigtail catheter, which is introduced by a majority of participating EPs, can clot, and in a worst-case scenario, the pigtail has to be exchanged. This is facilitated over the sheath as a continuous and safe access to the pericardial space. Of note, in tall or obese patients, it might be beneficial to use a longer or even a transseptal sheath.

Haemostasis and anticoagulation play a major role in the acute treatment of PT. Protamine administration in order to antagonize previously applied heparin in left atrial/left ventricular procedures is an essential step. Particularly in centres without institutional cardiac surgery backup, early application of protamine is the strategy of choice. However, the administration of protamine also bears a risk for clot formation inside the pericardial space, which can complicate the situation by impeding further and complete drainage of the pericardial effusion. This is probably the reason why about one-third of responders decide to first aspirate all blood from the pericardium before protamine is administered. In ∼80%, the dose of protamine depends on previously measured ACT levels or the total dose of applied heparin.

Additional application of clotting factors or NOAC antidotes is not performed by the majority of responders. While clotting factors such as prothrombin complex concentrate, prothrombin complex, or fresh frozen plasma are considered only by 8.3%, NOAC antidotes are routinely applied by only 15.2%. The decision to administer NOAC antidotes might be influenced by costs but also by the fact that PT even in patients under NOAC therapy might be safely and effectively managed without antidotes.

Auto-transfusion of aspirated blood is an important but disputatious aspect in PT management. Its potential advantages are immediate use, easy implementation, low costs, and avoidance of volume and blood loss, and thus, there is mostly no need for donor blood transfusion. Accordingly, 74% of all participants perform re-transfusion of aspirated blood, and almost three-thirds do it directly and without a mechanical blood filter. About 16% use a blood filter and 12% would re-transfuse via a cell safer. However, the latter is time-consuming to prepare and is therefore often not practicable in an emergency situation. Of note, 90% report to not have a fixed volume limit and would re-transfuse aspirated blood as long as necessary and reasonable.

Involvement of surgical backup and repair is also a controversial issue. Centres having an institutional cardiac surgery may have more leeway, since the decision for surgical repair can be taken anytime if the patient’s condition demands. However, if there is no institutional cardiac surgery but rather external cardiac surgical cooperation partners, the decision for potential surgical repair might be taken at earlier stages of the treatment cascade. The important aspect is to have cardiac surgical backup that is permanently available or on demand. The point in time to decide for surgical intervention is certainly a very individualistic decision to take. In our survey, 56% stated to involve surgeons if bleeding continues for more than 60–80 min, while another 25% would do if the total amount of aspirated blood would exceed an amount of up to 3000 mL.

### Post-interventional aspects

Keeping the pigtail catheter in place for several hours after successful treatment of PT is a double-edged weapon.^19^ First, further drainage from the pericardium might be necessary in case of ongoing or recurring bleeding. On the other hand, pericarditis might develop if the pigtail catheter is kept and patients normally complain about thoracic discomfort. In the survey, almost 50% of respondents state to keep the pigtail catheter until there is no evidence of further bleeding after re-initiation of an indicated anticoagulation. There are different types of medical strategies following pericardial puncture and drainage aiming mainly for pain relief and prevention of pericarditis including NSAIDs and colchicine applied by almost 50% of responders each. Also, antibiotics are applied by more than 30% of responders, but mostly for only 2 days.

The decision for re-initiation of an indicated oral anticoagulation after pigtail removal has to be carefully taken. On the one hand, left atrial thrombus formation and potential ischaemic stroke need to be prevented, and on the other hand, there would be the risk of ongoing or recurrent bleeding. With 0–72 h, there is a broad window within which anticoagulation is re-initiated by respondents.

### Onsite cardiac surgery vs. no onsite cardiac surgery

Having onsite cardiac surgery may affect not only the spectrum of EP procedures that is performed but also the mode of treatment in case of a PT. While many parameters of our analysis given by the participants are comparable, there are also differences: in centres with onsite cardiac surgery, the mean number of performed procedures is higher. Although epicardial VT ablation is frequently offered at centres with onsite cardiac surgery, the number of participants reporting on epicardial VT ablation without having onsite cardiac surgery is considerably high, with a rate of 37%. This finding is of interest when considering the ongoing debate on whether a procedure with a rather high incidence of major complications such as severe PT should be offered and performed at such centres. Moreover, protamine is less often applied by participants at onsite cardiac surgery centres. In centres without cardiac surgery, usually all efforts are taken to stop bleeding as soon as possible, and thus, protamine might be applied at earlier stages of the treatment cascade.

### Limitations

The analysed data in this study are based on per physician and not per centre level, and thus, an overestimation of the perspectives of large EP centres cannot be ruled out. The voluntary nature of the survey favours selection bias and raises questions whether these results represent a realistic reflection of the current practice. The survey included a limited number of only 26 questions. Therefore, further details such as incidences of PT or the need for surgical intervention could not be provided.

## Conclusions

The current survey demonstrates that the management of cardiac tamponade differs among EP centres. The findings of this survey may help to guide operators in their treatment and decision-making in the setting of PT.

## Supplementary Material

euad378_Supplementary_Data

## Data Availability

All relevant data are within the manuscript and its Supplementary material online files.

## References

[1] Hindricks G, Potpara T, Dagres N, Arbelo E, Bax JJ, Blomström-Lundqvist C et al 2020 ESC guidelines for the diagnosis and management of atrial fibrillation developed in collaboration with the European Association for Cardio-Thoracic Surgery (EACTS): the task force for the diagnosis and management of atrial fibrillation of the European Society of Cardiology (ESC) developed with the special contribution of the European Heart Rhythm Association (EHRA) of the ESC. Eur Heart J 2021;42:373–498. 32860505 10.1093/eurheartj/ehaa612

[2] Cappato R, Calkins H, Chen SA, Davies W, Iesaka Y, Kalman J et al Updated worldwide survey on the methods, efficacy, and safety of catheter ablation for human atrial fibrillation. Circ Arrhythm Electrophysiol 2010;3:32–8. 19995881 10.1161/CIRCEP.109.859116

[3] Deshmukh A, Patel NJ, Pant S, Shah N, Chothani A, Mehta K et al In-hospital complications associated with catheter ablation of atrial fibrillation in the United States between 2000 and 2010: analysis of 93 801 procedures. Circulation 2013;128:2104–12. 24061087 10.1161/CIRCULATIONAHA.113.003862

[4] Cheng EP, Liu CF, Yeo I, Markowitz SM, Thomas G, Ip JE et al Risk of mortality following catheter ablation of atrial fibrillation. J Am Coll Cardiol 2019;74:2254–64. 31672181 10.1016/j.jacc.2019.08.1036

[5] Fink T, Sciacca V, Feickert S, Metzner A, Lin T, Schlüter M et al Outcome of cardiac tamponades in interventional electrophysiology. Europace 2020;22:1240–51. 32500141 10.1093/europace/euaa080

[6] Bollmann A, Ueberham L, Schuler E, Wiedemann M, Reithmann C, Sause A et al Cardiac tamponade in catheter ablation of atrial fibrillation: German-wide analysis of 21 141 procedures in the Helios atrial fibrillation ablation registry (SAFER). Europace 2018;20:1944–51. 29982554 10.1093/europace/euy131

[7] Chierchia GB, Capulzini L, Droogmans S, Sorgente A, Sarkozy A, Müller-Burri A et al Pericardial effusion in atrial fibrillation ablation: a comparison between cryoballoon and radiofrequency pulmonary vein isolation. Europace 2010;12:337–41. 20056597 10.1093/europace/eup422

[8] Yang E, Ipek EG, Balouch M, Mints Y, Chrispin J, Marine JE et al Factors impacting complication rates for catheter ablation of atrial fibrillation from 2003 to 2015. Europace 2017;19:241–9. 28172794 10.1093/europace/euw178

[9] Doldi F, Geßler N, Anwar O, Kahle AK, Scherschel K, Rath B et al In-hospital mortality and major complications related to radiofrequency catheter ablations of over 10 000 supraventricular arrhythmias from 2005 to 2020: individualized case analysis of multicentric administrative data. Europace 2023;25:130–6. 36006798 10.1093/europace/euac146 PMC10103566

[10] Ekanem E, Reddy VY, Schmidt B, Reichlin T, Neven K, Metzner A et al Multi-national survey on the methods, efficacy, and safety on the post-approval clinical use of pulsed field ablation (MANIFEST-PF). Europace 2022;24:1256–66. 35647644 10.1093/europace/euac050 PMC9435639

[11] Schmidt B, Bordignon S, Neven K, Reichlin T, Blaauw Y, Hansen J et al EUropean real-world outcomes with Pulsed field ablatiOn in patients with symptomatic atRIAl fibrillation: lessons from the multi-centre EU-PORIA registry. Europace 2023;25:euad185. 37379528 10.1093/europace/euad185 PMC10320231

[12] Eckardt L, Doldi F, Anwar O, Gessler N, Scherschel K, Kahle AK et al Major in- hospital complications after catheter ablation of cardiac arrhythmias—individual case analysis of 43,031 procedures. Europace 2023;26:euad361. 38102318 10.1093/europace/euad361 PMC10754182

[13] Darden D, Aldaas O, Du C, Munir MB, Feld GK, Pothineni NVK et al In-hospital complications associated with pulmonary vein isolation with adjunctive lesions: the NCDR AFib Ablation Registry. Europace 2023;25:euad124. 37184436 10.1093/europace/euad124 PMC10228609

[14] Darma A, Dinov B, Bertagnolli L, Torri F, Lurz JA, Dagres N et al Cardiac tamponade complicating ventricular arrhythmia ablation: real life data on incidence, management, and outcome. J Cardiovasc Electrophysiol 2023;34:403–11. 36434796 10.1111/jce.15760

[15] Metzner A, Reubold SD, Schönhofer S, Reißmann B, Ouyang F, Rottner L et al Management of pericardial tamponade in the electrophysiology laboratory: results from a national survey. Clin Res Cardiol 2023;112:1727–37. 35713695 10.1007/s00392-022-02042-x PMC10697891

[16] Wasmer K, Zellerhoff S, Köbe J, Mönnig G, Pott C, Dechering DG et al Incidence and management of inadvertent puncture and sheath placement in the aorta during attempted transseptal puncture. Europace 2017;19:447–57. 27001035 10.1093/europace/euw037

[17] Chen H, Fink T, Zhan X, Chen M, Eckardt L, Long D et al Inadvertent transseptal puncture into the aortic root: the narrow edge between luck and catastrophe in interventional cardiology. Europace 2019;21:1106–15. 30887036 10.1093/europace/euz042

[18] Mathew S, Feickert S, Fink T, Rillig A, Reissmann B, Rottner L et al Epicardial access for VT ablation: analysis of two different puncture techniques, incidence of adhesions and complication management. Clin Res Cardiol 2021;110:810–21. 32719917 10.1007/s00392-020-01711-z PMC8166684

[19] Zhao Q, Li L, Liu N, Zhang M, Wu K, Ruan Y et al Early versus delayed removal of the pericardial drain in patients with cardiac tamponade complicating radiofrequency ablation of atrial fibrillation. J Cardiovasc Electrophysiol 2020;31:597–603. 31904158 10.1111/jce.14332

