# Supplementary material for: Prevention and treatment of pericardial tamponade in the electrophysiology laboratory: a European Heart Rhythm Association survey
Source: Europace. 2024 Jan 2;26(1):euad378. doi: 10.1093/europace/euad378 (PMC10787481; doi:10.1093/europace/euad378)
Supplement: euad378_Supplementary_Data [file euad378_supplementary_data.pdf]

EHRA European Heart Rhythm Association

Tamponade-Survey

Introduction

Dear colleagues,
Despite continuous efforts to improve the safety of catheter ablation, cardiac tamponade is still one of the most frequent and potentially life-threatening complications in the EP-lab. However, management of cardiac tamponade is not standardized and several uncertainties remain such as heparin-antagonization, modalities of auto-transfusion of aspirated blood or timing of cardiac surgery.
This survey evaluates the management of cardiac tamponade in ablation centers and focuses on precautions, periprocedural and postprocedural management of cardiac tamponade.

* 1. GDPR Disclaimer

We will not disclose your identity to any third party.

We comply with the European General Data Protection Regulation (GDPR) 2016/679. Any personal data processed in connection with this survey will be treated confidentially and only used by the ESC for the purposes of market research and not for promotion. Survey results will be kept for a maximum of 48 months for analysis and quality control purposes. We take all reasonable care to prevent any unauthorised access to your personal data. We respect your privacy and your right to access, modify, or remove your personal data. At any time, you can ask to know what personal data is being held. If you have any questions about data protection or require further information, please contact our data protection officer (DPO) at dpo@escardio.org.

You have the right to end your participation in this survey at any time.

Please confirm that you have read the above and agree to participate in this survey.

○ yes

○ no

EHRA European Heart Rhythm Association

Tamponade-Survey

Baseline institutional data

2. In Which country do you practice?

[ ]

3. In which hospital type do you work?

- ◯ Academic
- ◯ Non-academic
- ◯ Private
- ◯ Other (please specify)

4. How many EP-procedures are performed at your centre per year?

Diagnostic EP-procedures

Ablation of Supraventricular Tachycardia (SVT)

Ablation of Atrial Fibrillation (AF)

Ablation of Atrial Flutter/Atrial Tachycardia

Ablation of Ventricular Tachycardia (VT)

Epicardial VT-ablation

Occlusion of Left Atrial Appendage (LAA)

EHRA European Heart Rhythm Association

Tamponade-Survey

Infrastructure/Safety precautions:

5. Do you have a dedicated EP-lab?

- ◯ Yes
- ◯ No

6. Which mapping and/or ablation systems are applied at your centre? (Please indicate all modalities)

- [ ] Radiofrequency current - conventional
- [ ] Radiofrequency current with 3D-mapping and Contact Force
- [ ] Radiofrequency current with 3D-mapping, no Contact Force
- [ ] Cryoballoon
- [ ] Pulsed field ablation
- [ ] Other (please specify)

7. Do you have onsite cardiac surgery?

- ○ Yes
- ○ No

8. Do you have echocardiography permanently available inside the EP-lab?

- ○ Yes
- ○ No

9. Do you have a special epicardial puncture set prepared for emergency situations?

- ○ Yes
- ○ No

10. Do you have any restrictions and/or limits for ablation procedures at your centre for BMI?

- ○ yes, for left atrial/-ventricular procedures
- ○ yes, for all procedures
- ○ no

11. Is yes please provide the BMI-limit

yes, for left atrial/-ventricular procedures

yes, for all procedures

12. Do you have any restrictions and/or limits for ablation procedures at your centre for age?

- ○ yes, for left atrial/-ventricular procedures
- ○ yes, for all procedures
- ○ no

13. If yes, please provide the age-limit

yes, for left atrial/-ventricular procedures	
yes, for all procedures	

14. Do you have any restrictions and/or limits for ablation procedures at your centre for INR-value?

- ◯ no
- ◯ yes, INR-limit

15. Do you stop NOAC therapy before the procedure?

- ◯ yes, the day before
- ◯ yes, the evening before
- ◯ yes, at the day of the procedure
- ◯ no
- ◯ other strategies

16. In which of the following procedures do you perform invasive blood pressure monitoring?

- ☐ diagnostic EP procedures
- ☐ SVT-ablation
- ☐ AF-ablation
- ☐ Atrial flutter-/Atrial Tachycardia ablation
- ☐ VT-ablation
- ☐ LAA-occulder implant
- ☐ none

17. Which imaging modalities other than fluoroscopy do you use to guide the transseptal puncture?

- ☐ Transoesophageal echocardiography (TOE)
- ☐ Intracardiac echocardiography (ICE)
- ☐ none
- ☐ Other (please specify)

18. Which diagnostic catheter(s) do you use to guide your transseptal puncture?

- [ ] Coronary sinus (CS)-catheter
- [ ] His-catheter
- [ ] Pigtail/wire in the aorta
- [ ] none

19. What additional modalities do you use for guidance of transseptal puncture?

- [ ] Pressure control
- [ ] Contrast staining of the fossa ovalis
- [ ] Introduction of guidewire after transseptal puncture/access
- [ ] Injection of contrast after transseptal puncture/access
- [ ] Other (please specify)

EHRA European Heart Rhythm Association

Tamponade-Survey

Treatment of pericardial tamponade

20. Which imaging modalities do you use for emergency pericardial puncture?

- [ ] fluoroscopy in RAO
- [ ] fluoroscopy in LAO
- [ ] fluoroscopy in AP
- [ ] echocardiography
- [ ] none
- [ ] Other (please specify)

21. Do you intend an anterior or posterior epicardial access?

- ( ) anterior
- ( ) posterior

22. After successful epicardial access, do you introduce/place a sheath into the pericardium?

- ◯ Yes of 5F
- ◯ Yes of 6F
- ◯ Yes of 7F
- ◯ Yes of 8F
- ◯ No
- ◯ Other (please specify)

23. What pigtail catheter size do you use?

- ◯ 5F
- ◯ 6F
- ◯ 7F
- ◯ Other (please specify)

24. Do you apply protamin in the setting of a pericardial tamponade?

- ◯ Yes
- ◯ No

25. if yes, when?

- ◯ as soon as a pericardial tamponade is diagnosed
- ◯ after safe access to the pericardium is established
- ◯ after all blood is aspirated from the pericardium
- ◯ Other (please specify)

26. How much protamin do you apply?

- ◯ 3000 I.E.
- ◯ 5000 I.E.
- ◯ according to previously applied heparin in 1:1 ratio
- ◯ depending on last measured ACT level
- ◯ Other (please specify)

27. Do you use NOAC antidotes in case of a pericardial tamponade when NOAC-therapy was continued?

- Yes
- No
- only in the following situation

28. Do you regularly apply clotting factors (PPSB, aPPSB, recombinant FVIIa) in the setting of a pericardial tamponade?

- Yes
- No
- If yes, what kind

29. Do you retransfuse/autotransfuse blood which is aspirated from the pericardium?

- yes, but only before protamin administration
- yes, also after protamin administration
- Only if pericardial effusion cannot be controlled
- no
- Other (please specify)

30. if yes, how

- without blood filter
- with blood filter
- with cellsafer
- Other (please specify)

31. Do you have a maximal limit of retransfusion/autotransfusion?

- Yes
- No
- Other (please specify)

32. How and when do you decide for surgical intervention after all conventional measures?

in case of continuous pericardial bleeding after (min) _______________

after a minimal amount of _______________ml of aspirated blood regardless of bleeding time

Other (please specify)

Postinterventional treatment:

33. When do you remove the pigtail catheter or drainage system?

- immediately, if the bleeding has stopped
- if there is no further bleeding after re-initiation of an indicated anticoagulation
- Other (please specify)

34. After pericardial tamponade, do you routinely treat your patients with (please indicate for how many days)

NSAR (for ______days)

colchicin (for ______days)

cortison (for ______days)

antibiotics (for ______days)

Other (please specify)

35. After the pericardial effusion has stabilized, when do you restart an indicated NOAC therapy

after ______ hours

______ hours after removal of the pericardial drainage

Other (please specify)

Incidence of pericardial tamponade

36. How many pericardial tamponades have occurred at your centre over the last 12 months?

0 100

EHRA European Heart Rhythm Association
Tamponade-Survey

Thank you

Dear colleague,

Thank you very much for completing the survey.

Your input is very much appreciated and will provide us with invaluable insights.

Please click on “Done” to submit your responses.
